# Are children of key population individuals at higher risk of HIV than other children? Results from a multi-country analysis of routine program data

**DOI:** 10.1371/journal.pone.0309847

**Published:** 2024-09-06

**Authors:** Caterina Casalini, Sarah Yeiser, Hanna Amanuel, Amy Gottlieb, Chris Akolo, Madje Koffivi Toovi, Dismas Gashobotse, Pablo Mabanza, Bernard Ogwang, Sandra Georges, Natasha Mack, Meena Srivastava

**Affiliations:** 1 FHI 360, HIV Department, Washington, DC, United States of America; 2 Global Health Security and Diplomacy-U.S. President’s Emergency Plan for AIDS Relief, formerly at United States Agency for International Development, Washington, DC, United States of America; 3 FHI 360, HIV Department, Lomé, Togo; 4 FHI 360, HIV Department, Bujumbura, Burundi; 5 FHI 360, HIV Department, Lubumbashi, Democratic Republic of the Congo; 6 FHI 360, HIV Department, Dar es Salaam, Tanzania; 7 FHI 360, HIV Department, Abidjan, Côte d’Ivoire; 8 FHI 360, Scientific, Technical, and Evidence Advancement, Durham, NC, United States of America; University of the Witwatersrand Johannesburg Faculty of Health Sciences, SOUTH AFRICA

## Abstract

**Introduction:**

Children of key population individuals (CPK) often face the same stigma and discrimination as their parents, limiting their access to HIV services. The Meeting Targets and Maintaining Epidemic Control project analyzed pediatric HIV testing data from project-supported sites to better understand risk among CKP and improve comprehensive prevention, testing, and treatment for KP families.

**Methods:**

We conducted a retrospective analysis of routine program data collected October 1, 2021–September 30, 2022, in project-supported sites in Burundi, Côte d’Ivoire, Democratic Republic of Congo, Tanzania, and Togo. We compared HIV case finding (defined as the percentage of children diagnosed with HIV among those who were tested) and treatment initiation (defined as the percentage of children diagnosed with HIV who were initiated on antiretroviral therapy) data for children <15 years disaggregated by index versus non-index testing and CKP versus children of non-KP individuals (non-CKP).

**Results:**

A total of 5,651 children were tested (n = 2,974 index testing; n = 2,677 non-index testing). Of those diagnosed with HIV, 33% (181/541) were CKP, with case finding 17% (181 positive/1,070 tested) among CKP and 8% among non-CKP (360 positive/4,581 tested). Almost half of CKP diagnosed were ages 1–4 years. Among the 2,974 (53%) reached through index testing, overall case finding was higher among CKP (17%; 178 positive/1,052 tested) than non-CKP (11%; 219 positive/1,922 tested). Treatment initiation was 97% among CKP and 94% among non-CKP.

**Discussion:**

CKP were identified primarily through index testing which, although considered a priority strategy to identify children at high risk, has not been widely used within KP family networks. Most CKP reached were children of female sex workers, but those of other KPs should also be prioritized.

**Conclusions:**

KP-focused programs have often excluded children, but the case-finding approaches in the project’s KP programs were effective in reaching CKP. Comprehensive, family-centered KP programming is needed that includes family planning, prevention of vertical transmission, early infant diagnosis, and other maternal and child health services to reduce the impact of HIV on families and achieve an HIV-free generation.

## Introduction

The children of key population (KP) individuals living with HIV often face the same stigma and discrimination experienced by their parents, creating an added layer of vulnerability that limits their access to HIV services [1, 2]. Of the 1.5 million children under age 15 years living with HIV globally, only half (57%) were on life-saving antiretroviral therapy (ART) in 2022 [3]. Given this large pediatric treatment gap, case-finding approaches are needed to maximize coverage, particularly for the biological children of people living with HIV (PLHIV), including the children of KP individuals (CKP) who are living with HIV.

The Joint United Nations Programme on HIV/AIDS (UNAIDS) defines KPs as men who have sex with men (MSM), transgender (trans) people, sex workers, people who inject drugs (PWID), and people in prisons or other closed settings [4]. Although KP individuals account for less than 5% of the global population, in 2021, UNAIDS estimated that KP individuals and their sexual partners comprised 70% of new HIV infections globally and 51% of new HIV infections in sub-Saharan [5] Africa. Given this, the children of KP individuals living with HIV have an elevated risk of HIV infection and warrant particular focus in HIV service programming [6, 7].

However, programming that reaches KP individuals living with HIV who are parents frequently does not incorporate or provide specific services to address the needs of their children; as a result, HIV testing uptake among these children is low and linkage to treatment initiation and other health services is weak [8]. In recognition of this gap, in its 2022 strategy, the U.S. President’s Emergency Plan for AIDS Relief (PEPFAR) articulated a strategic direction focusing on equitable access to HIV services among priority populations—adolescent girls and young women, children, and KPs. The strategy intentionally prioritizes these groups for accelerated access to evidence-based HIV prevention and treatment programming [9].

The Meeting Targets and Maintaining Epidemic Control (EpiC) project offers HIV testing services (HTS), including safe and ethical pediatric index testing, to at-risk CKP and children of non-KP individuals (non-CKP) to improve case identification and access to antiretroviral therapy (ART) in several countries. We analyzed pediatric HIV testing data from EpiC-supported sites in five countries to better understand risk among CKP and improve comprehensive prevention, testing, and treatment support for KP individuals and their children.

## Materials and methods

### Design

We conducted a retrospective analysis of aggregate program data collected October 1, 2021–September 30, 2022, as part of routine monitoring in EpiC-supported sites in five sub-Saharan African countries. The data were extracted from an existing project database for HTS sites reporting HIV testing and treatment data for children under age 15 and which disaggregated them by CKP and non-CKP.

### Setting and population

EpiC-supported regions in five countries were selected for the analysis based on their reporting of testing data for CKP: Burundi (seven regions), Côte d’Ivoire (13 regions), Democratic Republic of Congo (DRC) (three regions), Tanzania (eight regions), and Togo (two regions). The data were for children and adolescents <15 years of KP and non-KP parents of any HIV status. CKP were defined as children of female sex workers (FSWs), and other KP groups, such as male sex workers (MSWs), MSM, PWID, or trans people, while non-CKP were defined as children whose parents/caregivers did not self-report as KP individuals.

In addition, index testing data for the KP parents were disaggregated by KP typology: FSW versus “other KP.” We conducted a sub-analysis of the index testing cascade of FSWs living with HIV to present the number of FSWs living with HIV to whom index testing was offered and who accepted index testing for their biological children, and the number of biological children who were elicited and tested. We did not have the data to conduct the same index testing sub-analysis for other KP parents. In addition, we did not have data on KP parent group for CKP who were tested through non-index testing.

### HIV testing intervention

The project offered HTS for both CKP and non-CKP via index testing (for biological children of an HIV-positive parent) and non-index testing (for children not identified as having a known HIV-positive biological parent) in facility and community settings. Of note, non-index testing results were only reported by two of the five countries, Côte d’Ivoire and DRC.

For index testing at facilities, health workers proposed that KP and non-KP parents testing positive for HIV voluntarily list their biological children, among other contacts. The contacts were then offered HTS. Both types of testing also reached CKP and non-CKP at antenatal clinics through pregnant women living with HIV.

One approach for non-index testing was provider-initiated testing and counseling (PITC) following administration of a risk-assessment tool, which varied by country but generally focused on malnutrition, hospitalization, and growth delay. These facility settings included outpatient, inpatient, malnutrition, and tuberculosis clinics.

In addition, both index and non-index testing were conducted in the community. One way was via orphans and vulnerable children (OVC) programming, where OVC case managers identified and referred CKP and non-CKP to HTS counselors offering mobile testing. CKP were also reached by KP peer navigators in the community through their parents. For example, FSW peer navigators reached and referred children of FSWs living with HIV to the KP-led drop-in center for HTS or, less frequently, to the public health facility. Non-CKP were also reached in communities through other peer outreach workers who helped navigate them to community testing sites.

ART was offered for all children found positive for HIV. Treatment was initiated both at sites supported by EpiC and those EpiC did not support, depending on family needs and preferences. To facilitate linkage to services, peer-led navigation to ART sites was offered to children and their caregivers.

### Data collection and quality assurance

Providers certified in HTS and ART collected the data using national and project tools. Client information was entered into paper-based national tools, and data entry clerks reviewed data for completeness and consistency before entering them into the national health management information system (HMIS) and project database. All data were regularly validated through the project’s established processes for data quality assurance using data triangulation between the paper tools and electronic database. Built-in validation rules within the database mitigated data entry and transcription errors. A gap analyzer was run on the database to identify data errors, which were then checked against the source documents and cleaned. Any gaps and outliers identified were reconciled prior to reporting. All aggregated, de-identified data were managed by the project strategic information team.

We analyzed HIV testing data (disaggregated by index versus non-index testing) and treatment initiation data for CKP and non-CKP. We also conducted a sub-analysis of the index testing cascade of FSWs living with HIV and their biological children; as noted, we did not have the data to conduct a sub-analysis of CKP from other parent KP groups tested through index testing.

### Data analysis

The routinely collected data for the period of analysis (October 1, 2021–September 30, 2022) were disaggregated by age band, testing modality, testing venue (facility versus community) and type of child (CKP versus non-CKP). We compared data from baseline (the quarter October 1–December 31, 2021) to endline (the quarter July 1–September 30, 2022) for the following indicators: HIV testing (number of children who received HTS), HIV positive (number of children who received a positive HIV test result from the HTS), HIV case-finding rate (percentage of children diagnosed with HIV among those who were tested), and treatment initiation rate (percentage of children diagnosed with HIV at EpiC-supported sites who were initiated on ART at both EpiC- and non-supported sites at any point after diagnosis). In addition, we conducted an index testing sub-analysis for FSWs living with HIV and their biological children, for which we calculated the FSW offer rate (defined as the percentage of FSWs living with HIV to whom index testing of their biological children was offered), the FSW acceptance rate (defined as the percentage of FSWs living with HIV who accepted index testing of their biological children), the testing rate of FSWs’ biological children (defined as the percentage of elicited biological children of FSWs living with HIV who were tested for HIV), and the case-finding rate for FSWs’ biological children (defined as the percentage of biological children living with HIV among those who were tested).

### Ethical considerations

The Protection of Human Subjects Committee at FHI 360 made a non-human-subjects research determination for analyses of EpiC routine data on May 28, 2021. The routine program data were extracted from the project database on July 5, 2023. Only aggregated program data were analyzed, and at no time did the authors have access to individual-level data or personally identifiable information.

In the EpiC-supported program activities for which the data were collected, all guidelines were followed for safe and ethical index testing, including for KP individuals and their partners and children [10]. All children were tested following consent from the biological parent: Parental consent was verbal if the child was physically present and written for biological children staying with another caregiver.

## Results

### Overall CKP and non-CKP results

From October 1, 2021, through September 30, 2022, 5,651 children received HTS at the project-supported sites across the five countries, of whom 19% (1,070/5,651) were CKP (Table 1). Overall, the project identified 541 children living with HIV (10% case finding). A total of 181 (33% of all children tested) were CKP and 360 (67% of all children tested) were non-CKP. Despite lower numbers of CKP reached, they had higher case-finding rates than non-CKP in all five countries (17% case finding versus 8% case finding, respectively). Tanzania and DRC accounted for 69% of the total number of children tested across the five countries and 85% of the CKP diagnosed with HIV; DRC reported the highest case-finding rate for CKP through index testing, at 33%. The ART initiation rate was 95% overall: 97% among CKP and 94% among non-CKP.

**Table 1:** HIV testing (Index and Non-index) and ART initiation among CKP and non-CKP by country, October 1, 2021–September 30, 2022.

Country	Burundi		Côte d’Ivoire		DRC		Tanzania		Togo		All Countries		Total
Indicators	Non-CKP	CKP	Non-CKP	CKP	Non-CKP	CKP	Non-CKP	CKP	Non-CKP	CKP	Non-CKP	CKP	
**Index tested**	0	35	113	106	989	227	820	508	0	176	1,922	1,052	2,974
**Index tested positive**	0	4	5	16	140	76	74	75	0	7	219	178	397
**Index case finding**		11%	4%	15%	14%	33%	9%	15%	-	4%	11%	17%	13%
**Non-index tested**	0	0	106	13	2,511	5	42	0	0	0	2,659	18	2,677
**Non-index tested positive**	0	0	2	1	139	2	0	0	0	0	141	3	144
**Non-index case finding**	-	-	2%	8%	6%	40%	0%	-	-	-	5%	17%	5%
**Total tested**	0	35	219	119	3,500	232	862	508	0	176	4,581	1,070	5,651
**Total tested positive**	0	4	7	17	279	78	74	75	0	7	360	181	541
**Total case finding**	-	11%	3%	14%	8%	34%	9%	15%	-	4%	8%	17%	10%
**ART initiation**	0	4	8	16	254	75	75	74	0	6	337	175	512
**Total ART initiation rate** ^*^	-	100%	114%	94%	91%	96%	101%	99%	-	86%	94%	97%	95%

^*^ART initiation rates higher than 100% reflect the initiation of children living with HIV who were diagnosed in previous reporting periods but initiated later; hence, they were not included among those who newly tested HIV positive.

### Index testing

Of the 5,651 children tested, 2,974 (53%) were reached through index testing, 397 of whom were diagnosed with HIV (13% case-finding rate). Ninety-eight percent (1,052/1,070) of all CKP and 42% (1,922/4,581) of all non-CKP tested were reached through index testing. Across the five countries, case finding from index testing was higher among CKP (17%) than among non-CKP (11%) and was likewise when analyzed by country (Table 1).

### Non-index testing

Of the 5,651 children tested, 2,677 (47%) were reached through non-index testing modalities, 144 of whom (5% case finding) were diagnosed with HIV. Of the children tested, 2% (18/1,070) of all CKP and 58% (2,659/4,581) of all non-CKP received HTS through non-index testing methods. No data were available on the parent KP groups for the 18 CKP tested through non-index testing methods. The number of CKP tested was smaller than for non-CKP, but case finding among CKP was higher at 17% versus 5% for non-CKP.

When analyzed by age group, case finding among CKP was 36% (4/11) for those younger than 1 year, 23% (79/342) for those 1–4 years, 13% (54/421) for children ages 5–9 years, and 14% (43/300) for 10–14-year-olds (Table 2). CKP <5 years accounted for 46% (83/180) and made up the largest proportion of all CKP diagnosed with HIV.

**Table 2:** CKP index testing volume and results by age group, all countries (Burundi, Côte d’Ivoire, DRC, Tanzania, Togo), October 1, 2021–September 30, 2022.

	<1 Year	1–4 Years	5–9 Years	10–14 Years	Total
**Tested**	11	342	421	300	1,074
**Tested positive**	4	79	54	43	180
**Case-finding rate**	36%	23%	13%	14%	17%

### Index testing sub-analysis of FSW parents living with HIV and their biological children

Testing data for the KP parents indicated FSWs as the largest number of KP individuals reached who also listed their biological children as index contacts. Among the 1,052 CKP tested through index testing, 936 (89%) were children of FSWs. The remaining 116 were children of other KP caregivers whose data were not available; therefore, it was not possible to conduct the index testing sub-analysis for these CKP.

The project reached 6,735 FSWs living with HIV, of whom 93% (6,248) were offered index testing of their biological children; 87% (5,408) of the FSWs living with HIV accepted the index testing of their biological children. A total of 1,357 biological children were elicited from these FSWs, of whom 936 (69%) were tested and 132 diagnosed with HIV (14% case finding) (data not shown). These children represented 89% of the total CKP tested and 74% of the CKP diagnosed with HIV through index testing.

## Discussion

During the analysis period (October 1, 2021–September 30, 2022), the EpiC-supported sites in Burundi, Côte d’Ivoire, DRC, Tanzania, and Togo tested 5,651 children of KP and non-KP parents living with HIV, among whom 541 were identified as living with HIV and initiated on treatment. About one-third of the children tested were CKP identified primarily through index testing. The other two-thirds tested were non-CKP, some of whom may have been the children of undisclosed KP partners, potentially explaining the relatively high case finding within this subpopulation.

Case finding among all children tested was high at 10%, indicating that the children at greatest risk were being reached. Case finding was twice as high for CKP as for non-CKP, suggesting not only the effectiveness of the program’s pediatric case-finding approaches but also the higher risk of HIV in this subpopulation of children. These findings reinforce the need for investments and focused efforts from donors and governments in case finding, timely diagnosis, and linkage to treatment for the biological children of KP individuals and other at-risk children.

The ART initiation rate was also high in both groups of children: 97% and 94% among CKP and non-CKP, respectively. This finding suggests that once CKP and non-CKP living with HIV were identified, the project-supported programs were able to successfully link them to ART services. Where allowed by national policy, ART initiation for CKP took place at drop-in centers authorized to provide pediatric care. For drop-in centers serving adults only, CKP diagnosed with HIV and their caregivers were offered peer-led navigation to public ART facilities. For CKP and non-CKP living with HIV identified at the facility level, the team from the ART clinic within the same facility was contacted and the child was usually started on ART the same day or within a maximum of one week. Non-CKP living with HIV identified at the community level were typically navigated by the project team to the health facility ART clinic on the following day.

Several studies have revealed the risk of HIV for children of FSWs, MSM, and PWID and the gaps in service uptake among these children. In a study from Côte d’Ivoire, although FSWs were engaged in HIV testing and prevention services, during their last pregnancy only 59% had received HIV testing before delivery and 30% had lost one or more child [7]. A study in South Africa found that nearly one-third of children of FSWs living with HIV had never received HIV testing [11], while in research in Cameroon, nearly 70% of FSWs living with HIV reported that none of their children had been tested for HIV before age 5 (326/481), and 3.5% (17/481) reported one or more of their children as having been diagnosed with HIV [7].

In our data, the majority of KP parents living with HIV were FSWs; however, the biological children of other KPs are also at risk. A study in India found that wives of MSM bear a high burden of HIV infection, with disclosed MSM and their wives having HIV prevalence of 46.9% and 27.5%, respectively. HIV prevalence among undisclosed MSM was 22.8%, but despite their wives being at high risk, they were unaware of their husband’s HIV status and less likely to engage in care [12]. Given this high HIV prevalence among married MSM, they are an important population for support, as wives and children are at high risk but likely have low risk perception [13, 14].

Consideration of risk among PWID and their children is also needed. In Northeast India, data collected from male (n = 5,653) and female (n = 796) PWID found significantly higher HIV prevalence among women than men (53% versus 18.4%, p<0.01), with 49% of men and 55% of women reporting being in a relationship (married or long-term partner) [15]. This points to a need for integrated HIV services for PWID to address factors at individual, interpersonal, family, and community levels.

Prevention of mother-to-child transmission (PMTCT) of HIV is a critical component of programming for KP women living with HIV. FSWs often have at least one biological child and commonly have low contraceptive use, high burden of unintended pregnancy, poor reproductive outcomes, and avoidable mother-to-child transmission risk [16]. Those living with HIV who are incarcerated have particularly limited access to PMTCT services [17]. A study in South Africa found that 77% of FSWs were mothers, of whom two-thirds were living with HIV. Unplanned pregnancies and late pregnancy discovery contributed to late presentation at antenatal care clinics and delayed ART initiation, increasing the risk of vertical transmission of HIV [18]. In the age-disaggregated data in our analysis, CKP <5 years accounted for the largest number and proportion of CKP diagnosed with HIV. This finding suggests a high rate of mother-to-child transmission and calls for urgent action to ensure that KP and clinical programs work together closely to prioritize and scale up PMTCT services for pregnant and breastfeeding KP women, including integrating family planning, HIV testing and maternal retesting, pre-exposure prophylaxis, and treatment services with active linkage to antenatal care. This has particular merit given the findings of a study in Burkina Faso indicating higher exposure to health care among FSWs seeking antenatal services [16]. Additionally, integrating antenatal care, PMTCT, early infant diagnosis, and other prevention and testing services into drop-in centers and through strong referral networks is needed for mothers and their HIV-exposed infants.

In this analysis, peer-led index testing was offered to almost all FSWs living with HIV, and although a large proportion (87%) accepted to elicit their contacts including biological children, only 69% children of FSW living with HIV were tested through index testing.

We do not have routinely collected data that might explain this, but it is possible that some children of FSWs living with HIV may have been living with other caregivers outside the project catchment area; and stigma and discrimination may have prevented them from accessing HTS. Mentor mothers have been recognized as playing major roles in promoting maternal health education, HIV testing and treatment, adherence, retention, and HIV disclosure for women living with HIV, as well as HIV counseling and testing of the children of women living with HIV [19]. To our knowledge, EpiC’s engagement of FSW mothers as peer navigators to reach peers and biological children is the only example of a mentor mother program specific to FSWs.

A review of the literature indicates that CKP experience extreme levels of vulnerability and risk in all core areas of care, development, and protection, including lack of a birth certificate, food insecurity and malnutrition, low school enrollment, poor access to essential health services, lack of childcare, experiencing physical and sexual violence, and stigmatization by families, neighbors, and other children in the community [8, 20–22]. These factors contribute to elevated HIV risk and gaps in case finding, linkage, treatment coverage, retention, and viral suppression for KP individuals and their children as seen in current programming. Marginalized KP individuals and their children are more likely to participate in HIV interventions, including OVC services, when the interventions are designed to meet their unique needs; supporting these children is an essential way to link and retain families in services.

However, recommendations for KP-focused HIV programming have often been adult-focused, excluding children and adolescents [23, 24]. To ensure health equity for KP individuals, their children, and partners/spouses, differentiated service delivery (DSD) approaches are fundamental. This requires bringing multiple stakeholders and sectors together [20, 22] and working collaboratively with family planning, PMTCT, OVC, and other maternal and child health services to improve the pediatric clinical cascade. Within this model, index testing, linkage to ART initiation, and retention in care should be implemented by trusted providers and peer networks, including with KP peers as mobilizers, navigators, and counselors, to build trust in and increase uptake of community-based HIV services [25]. In addition, service venues must be safe, acceptable, and friendly (e.g., KP-led drop-in centers), and the system designed to maintain the confidentiality of the HIV status of KP individuals and their children [26], as KP parents may fear discrimination by child protection workers and removal of children from the home. The EpiC program results are evidence for how the integration and differentiation of services for vulnerable CKP and their families can work.

### Limitations

Our analysis has several limitations. First, we only analyzed the FSW index testing cascade results because we did not have the index testing data of other KP parents or the non-index testing data for any KP parent group. As such, it was not possible to conduct additional sub-analyses; however, we acknowledge that these additional analyses are warranted to better understand the contribution of CKP to case finding by KP parent group. Another limitation is that we did not use a statistical method to compare CKP and non-CKP for index testing versus non-index testing. This was because 98% (1,052/1,070) of the CKP were reached through index testing and because the sample size for non-index testing among CKP was too small for statistical analysis.

## Conclusions

EpiC’s index testing and non-index testing approaches were effective in reaching children at high risk of HIV. Among the CKP reached with testing services, a high percentage were diagnosed with HIV, further highlighting that many of these children are at high risk and need a focused approach and more investment from donors and governments to ensure they are reached, identified, and linked to care and treatment in a timely manner.

Given the number of CKP in our analysis <5 years who were diagnosed with HIV, scale-up and strengthening of PMTCT and family planning services for KPs should be effected not only to prevent new child infections but also to ensure that pregnant and breastfeeding KP individuals and their children have equitable access to high-quality, life-saving health services.

## Supporting information

S1 Dataset FSW sub-analysis data. (XLSX)
